# Hospital Worthies

**Published:** 1886-11-13

**Authors:** S. L. Gulson

**Affiliations:** founder of the Coventry Children's Hospital


### Hospital Worthies.

Mrs. S. L. GULSON, Founder of the Coventry Children's Hospital.

Difficult to Commence.

Many people know by personal experience how difficult it is to start any new enterprise whatever. An indifferent public ridicules a new enterprise, and brands it as chimerical or absurd. Candid friends advise one to desist, foreseeing endless trouble and prophesying failure as a necessary result. If the enterprise entails a request for contributions of however small amount,—and experience leads us to conclude that the smaller the amount asked for the more difficulty there will be to collect it, then,—and probably only then, does the author of any novel scheme or undertaking, begin to realise the difference between the words "acquaintance" and "friend." In a country town which has been accustomed to one hospital, and one only, the inhabitants, not unnaturally perhaps, resent as an impertinence any proposal to start a new one for any class of cases whatever. Does not the existing institution provide accommodation for all, and does it not contain empty beds and shew a deficiency of income, which keeps its managers in a state of financial unrest at all times? We would not be understood as advocating the undue extension of special hospitals, but when the new institution is to provide accommodation for children, or cases of eye disease, in a country town, then there can be no question, that all the more enlightened residents should support it. Yet in this, as in all other human affairs, it is only too easy to be unconscious of neglect when it is sanctioned by custom. Whately once wrote, and wrote wisely, "It is to be observed that in almost every department of life, the evil that has very long existed will often be less clearly perceived, and less complained of, than in proportion to the actual extent of the evil. If you look to any department of government, or to any parish or diocese, that has long been left to the management of apathetic or inefficient persons, you will usually find that there are few or no complaints; because complaints, having been long since found vain, will have long since ceased to be made.

In 1874.

Twelve years ago Coventry had no special provision for the treatment of sick children, and the county hospital had not funds enough to furnish or maintain all its wards, much less to make special provision for the sick little ones. Any children who were so ill as to almost compel their admission, had to be treated in the adult wards. It is no reflection on the hospital committee to say, that this was not a satisfactory arrangement. It was not the best for adult patients, and it was not the best for sick children, who were necessarily placed under disadvantageous conditions, well calculated to retard their recovery. Obvious as were these facts, the financial depression, which crippled the county hospital, was held to be more than sufficient excuse for the committee who declared their inability to make any change. It is unfortunately true, that children are often deprived of the benefits of a hospital, by the laziness and apathy of their parents. Poor people do not always go to the doctor on account even of manifest and often serious illness, which, in their children at least, is often suffered to proceed unchecked with amazing indifference. In such circumstances, and we believe that they accurately describe the condition of affairs at Coventry, in regard to the provision of hospital accommodation for children in 1874, it clearly became the duty of some enlightened citizen, to step into the breach, as the champion of the children. This Mrs. Gulson did, and we hope many may be encouraged by this account of her successful efforts to go and do likewise.

What She Determined to do.

On inquiry, Mrs. Gulson found that some of the vacant wards could be isolated from the rest of the hospital, and so converted into a children's department or wing. Under these circumstances, she undertook to raise a fund sufficient to fit and furnish that portion of the Coventry Hospital now devoted to the children, and to guarantee to collect an income of £120 for the maintenance of the first ward, containing six beds, at an average cost of £20 each. To this was subsequently added a second ward, containing four beds, making ten in all, involving an annual outlay of £200. The wards were to be placed in charge of a special nurse, and were for all practical purposes to be detached from the rest of the hospital. No tickets were to be required for admission, but the fitness of applicants once tested by the hospital surgeons, and deserving cases were to be taken in at once. The children's wards, whilst under the same roof as the hospital, were to be maintained independently, and to be placed under separate management. The Committee, after due consideration, agreed to Mrs. Gulson's proposals, on condition that £200 per annum should be paid half-yearly in advance for the maintenance of the children's wing, and that no portion of the cost of furniture and maintenance should be borne by the ordinary revenue of the hospital. An agreement was drawn up and duly signed by Mrs. Gulson in 1874, and ever since then, she has undertaken every expense connected with the maintenance of the children's wards. Despite many anxieties and difficulties, she has never allowed the half-yearly contribution of £100 to be in arrear, but has always made this payment before it fell due.

How she did it.

Mrs. Gulson, having taken upon herself this heavy burden of responsibility, steadily devoted herself to the task of raising annually £200, and of collecting immediately several hundred pounds, in addition, to defray the cost of fitting up and furnishing the wards placed at her disposal for the children's hospital. She herself declares that she had great sympathy in her work, and that she was soon enabled to purchase the necessary fittings, to furnish the nurses' quarters, and to provide a bath-room, pantry, and other additions, so as to form a small and attractive children's hospital within the walls of the older institution. Notwithstanding Mrs. Gulson's modest assurance to the contrary, we know that in order to accomplish what she had undertaken, it became necessary for her to become possessed of this one idea, and to be ever and always striving to interest others in the sick children of Coventry. Knowing that wealthy people were few and difficult to approach, she decided to face the labour of awakening the interest of the many. With this object she started five-shilling annual subscriptions, and by this means she was enabled to raise the £200 per annum, that is to say, 800 people became interested in the children's hospital at Coventry, and regular subscribers to its funds, by the influence of one woman. Let anyone pause for a moment and try to realise how much labour must have been expended during the last twelve years in collecting the sum of five shillings annually from 800 separate people. If they do so, they must perforce agree, that such a practical illustration of the power for good possessed by one individual, who really exerted herself in the cause of her suffering neighbours, must inspire many to attempt good deeds which have before seemed to be almost, if not altogether, beyond the compass of the powers they possess.

New Developments

But Mrs. Gulson did not rest content when she had fitted up, furnished, and maintained the children's hospital. After four years' experience, despite the enormous amount of trouble and fatigue to all concerned in the in-getting of 800 five-shilling subscribers, she determined that the little hospital should never become extinct for want of funds to maintain it in efficiency. She felt that one day it would be impossible for her to any longer give the time and the energy necessary to raise the maintenance fund, and that then the children's hospital might fail for want of funds, and eventually disappear. Such a contingency was not to be thought of for a moment, and she determined to raise an endowment fund of £4,000 as an effectual preventive to any such disaster. Once resolved upon this new departure, Mrs. Gulson found, that the persistence with which the claims of the children's hospital had been kept before the public, had led some people to think, that too much fuss had been made about it. At this juncture the *Coventry Herald* came to the rescue, and pointed out, that the fuss complained of had had a very creditable and intelligible foundation, which had arisen from the necessity for finding the means of support for the children's wards, and the endowment fund appeal had the same justification. The *Herald* invited the public to visit the children's wards, to note the good order, cheerfulness, and thoughtful consideration by which all their arrangements were marked. A peep into the adults' wards, and a glance at their more staid occupants, would satisfy any visitor, that the division was not the result of mere caprice, but was justified by practical considerations affecting both classes of patients alike. For these reasons the work begun by Mrs. Gulson, and carried on under her unremitting supervision, was declared to be well worthy of the support, which could alone secure its permanent continuance. Thus aided, a bazaar was set on foot, and with its aid, and by written and other appeals, Mrs. Gulson had raised the sum of £2,722 by June 1883, which she has since increased to nearly £4,000. She has discovered, however, owing to the cheapness of money, that £4,000 will not bring in £200 a year, and has decided to endeavour to increase the endowment-fund to at least £4,500 by a second bazaar, which is to take place in Coventry on the 15th instant. A gratifying proof of the sympathy created by twelve years of devoted labour for suffering humanity is to be found in the fact, that Lady Brooke has consented to open the bazaar, and that it will be attended by the representatives of all classes, not only in Coventry, but throughout the county of Warwick. Can it be supposed that the men of Coventry will fail to be touched by such splendid devotion, or that there is the shadow of a shade of doubt, that the whole of the £4,500 at least will be forthcoming by the end of next week?

The Work finished, and After.

Mrs. Gulson has maintained ten cots for twelve years, which have been the means of affording relief to at least nine hundred or a thousand sick and suffering little ones, the children of the poor. Her work has been accomplished unostentatiously, but it has been thoroughly well done. She has had to write some fifteen hundred letters a year on behalf of her little charges, or, say, from fifteen to twenty thousand in all. Having borne the burden of this heavy charge for so long, she seems to cling with a mother's tenderness to this Coventry Nursery, where so many poor, helpless, and sick children, in the good providence of God, have found a renewed lease of life and health. It is evident that Mrs. Gulson could not bear the thought of the gradual disappearance of the little hospital, which might and probably would follow the eventual cessation of her efforts, when advancing years or failing health might render it impossible for her to continue at her post. In spite of the assistance of devoted friends, the aid of the local press, and the support derived from public sympathy, the labour involved from first to last during these twelve years upon the lady to whose exertions the Coventry Children's Hospital owes its existence, has been great indeed. Still, she has persevered with her work, and now that the endowment-fund is within £1,000 of its complement, who can withhold a contribution, as an act of sympathy with a good woman in a noble work courageously undertaken, and now all but completed? Who, indeed? But, when the endowment-fund is made up to the required sum, when the Coventry Children's Hospital is made a permanent institution by its safe investment, and Mrs. Gulson is at last freed from a heavy responsibility, and has the gratification of seeing the successful accomplishment of her life's work, something will yet remain to be done. Such good works as are here recorded ought to be kept in permanent remembrance for the encouragement and inspiration of those who come after. In Germany the Iron Cross, and in France the Legion of Honour, would be offered for Mrs. Gulson's acceptance. In England the first lady in the land has ever shown her sympathy with good deeds well and nobly done. In this year of the Queen's Jubilee, it is not impossible, therefore, that means may be found, to commemorate the establishment and permanent endowment of the Coventry Children's Hospital, in the person of its founder, Sophie Gulson.

